# Comparison of adipokines in a cross-sectional study with healthy overweight, insulin-sensitive and healthy lean, insulin-resistant subjects, assisted by a family doctor primary care program

**DOI:** 10.1186/s13098-016-0125-9

**Published:** 2016-02-09

**Authors:** Samuel D. Moscavitch, Hye C. Kang, Rubens A. C. Filho, Evandro T. Mesquita, Hugo C. C. F. Neto, Maria L. G. Rosa

**Affiliations:** Laboratory of Immunopharmacology, Oswaldo Cruz Institute (IOC), Avenue Brasil 4365, Manguinhos, Rio de Janeiro, 21045-900 Brazil; Pathology, Federal Fluminense University, Niteroi, Brazil; Clinical Medicine, Federal Fluminense University, Niteroi, Brazil; Epidemiology and Biostatistics, Federal Fluminense University, Niteroi, Brazil

**Keywords:** Adiponectin, Obesity, Lean, Insulin resistance, Inflammation, HOMA, BMI, Cytokine, C reactive protein, Adipokines

## Abstract

**Background:**

In most individuals, obesity and insulin resistance coexist. However, some individuals have excessive adipose tissue mass but remain insulin sensitive. Moreover, lean individuals can develop acute inflammation-induced insulin resistance, even without excess adipose tissue mass.

**Objective:**

Our aim was to compare inflammatory markers in overweight, insulin-sensitive and lean, insulin-resistant healthy subjects.

**Methods:**

A cross-sectional study with 1098 participants (CAMELIA project) was conducted in family doctor primary care program at Niteroi, RJ, Brazil. In the present substudy, we have selected non-obese healthy subjects (n = 203). Insulin resistance was defined by a homeostatic model assessment (HOMA-IR) >2.6, and overweight subject BMIs were 25< BMI <30 kg/m2. Associations were estimated through binary logistic regression with generalized estimation equation models.

**Results:**

We compared overweight, insulin-sensitive healthy individuals (n = 74) with a mean age of 39.2 ± 1.3 and lean, insulin-resistant healthy individuals (n = 18) with a mean age of 31.9 ± 3.6. C-reactive protein levels were positively correlated with body mass index in the lean, insulin-resistant group. In the multiple regression model, a positive association was observed with MCP-1 and IL-6 expression after adjustment for age, waist circumference, glycated hemoglobin, resistin, adiponectin, C-reactive protein and PAI-1 levels.

**Conclusion:**

Our findings suggest that a lean, insulin-resistant subject may have higher pro-inflammatory marker levels (MCP-1, IL-6 and resistin) than an overweight, insulin-sensitive subject. This suggest an early risk phenotype that should further be investigated for possible prognostic implications.

## Background

During the last century, epidemiologic studies have suggested a possible relationship between inflammation and insulin resistance [1]. More recently, this evidence has become robust and suggests that obesity and inflammation are the main components of insulin resistance [2, 3]. The adipose tissue is directly involved in the inflammatory response and produces several cytokines, such as adiponectin, resistin, interleukin-6 (IL-6), plasminogen activator inhibitor-1 (PAI-1) and monocyte chemotactic protein-1 (MCP-1). Excess adipose tissue also promotes inflammation, which is accompanied by monocyte and B and T lymphocyte infiltration [4]. Chronic inflammatory state in obesity is maintained by interactions between leptin and inflammation, where an increase on pro-inflammatory cytokines leads to leptin release. In most individuals, insulin resistance and obesity coexist. However, some subjects with excessive body fat have better insulin sensitivity than expected for their adiposity [5]. Conversely, lean subjects can develop inflammation-associated insulin resistance [6].

Despite the importance of inflammation, studies comparing the individual impacts of obesity and insulin resistance on inflammatory activation are not found in the literature. This comparison will expand our knowledge concerning the complex dynamics of inflammation, adiposity and insulin resistance.

Our aim was to describe and compare inflammatory markers in overweight, insulin-sensitive and lean, insulin-resistant subjects, assisted by a family doctors primary care program.

## Methods

The Cardio-neuro-Metabolic-renal Familial (CAMELIA) project is a transverse observational study with 1098 participants in which cardiologists, neurologists, psychiatrists, endocrinologists, nephrologists, general doctors, nutritionists, nurses and students participated; this project aimed to study cardiovascular risk factors and related issues, such as familial aggregation. This project was conducted between July 2006 and December 2007 in 13 modules of the Family Doctors Program of Niteroi, RJ, Brazil, which was selected in an attempt to include all of the politico-administrative regions of the city. Data collection (demographic, anthropometric, clinical, psychological, nutritional, and blood and urine samples) was performed during project visits to each regional family doctor clinic (to assess more detailed CAMELIA project information, see Ref. [7]). The insulin, PAI-1, MCP-1, resistin, adiponectin and IL-6 were analyzed on a Luminex^®^. C reactive protein (CRP) was analyzed using an ELISA commercial kit. The sample criteria selection for this substudy aimed to include healthy subjects, without any comorbidity. We included those who were older than 18 years; did not have any cardiovascular diseases (myocardial infarction, heart failure, cerebrovascular accident); were not under medication for hypertension, diabetes, or hyperlipidemia; had a BMI (body mass index) <30 kg/m^2^; and had no previous diagnosis of diabetes. Overweight was defined as having a BMI 25< BMI <30 kg/m^2^ [8]. Insulin resistance was defined as a homeostatic model assessment (HOMA-IR) >2.6 [9–11]. From among these subjects, we have selected 203 subjects and separated into 4 groups: (a) normal BMI subjects (<25 kg/m^2^) with preserved insulin sensitivity; (b) normal BMI subjects (<25 kg/m^2^) with insulin resistance (c) overweight subjects with preserved insulin sensitivity; (d) overweight subjects with insulin resistance. After analysis, we finally selected 92 non-obese healthy subjects and separated them into 2 groups: (I) normal BMI subjects (<25 kg/m^2^) with insulin resistance (n = 18), OW(−)IR(+); (II) overweight subjects with preserved insulin sensitivity (n = 74), OW(+)IR(−).

Parametric variables were analyzed with Student’s *t* test, and non-parametric variables were analyzed with the Spearman test correlation. Univariate parametric (Student’s T) and non-parametric (Mann–Whitney) tests were used to investigate potential differences between the groups. For generalized estimated equations (GEE), binary logistic regressions were performed with variables that reached p values <0.150 on parametric and non-parametric tests and included cytokines and variables related to adiposity and glucose metabolism. Statistical analysis was performed using SPSS Statistics 17^®^ software. The data were statistically significant at p < 0.05. The CAMELIA study protocol was approved by the Federal Fluminense University Ethics Committee (UFF/Huap#220/05), and all of the patients signed written consent forms.

## Results

**Fig. 1:**
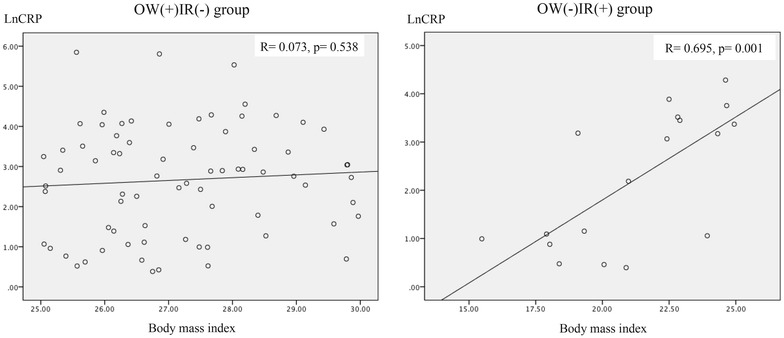
Correlation between C reactive protein and body mass index, according to the presence of overweight (n = 74) or insulin resistance (n = 18)

**Fig. 2:**
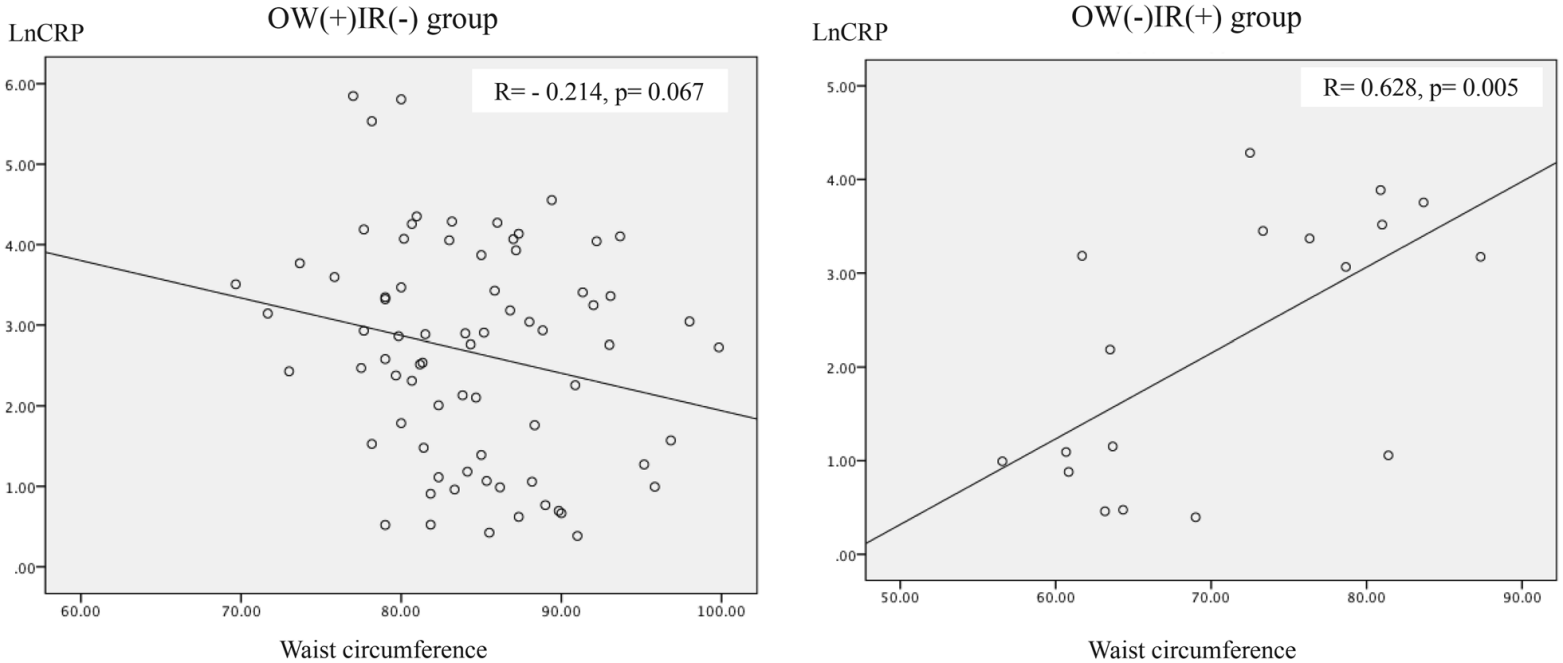
Correlation between C reactive protein and waist circumference, according to presence of overweight (n = 74) or insulin resistance (n = 18)

**Fig. 3:**
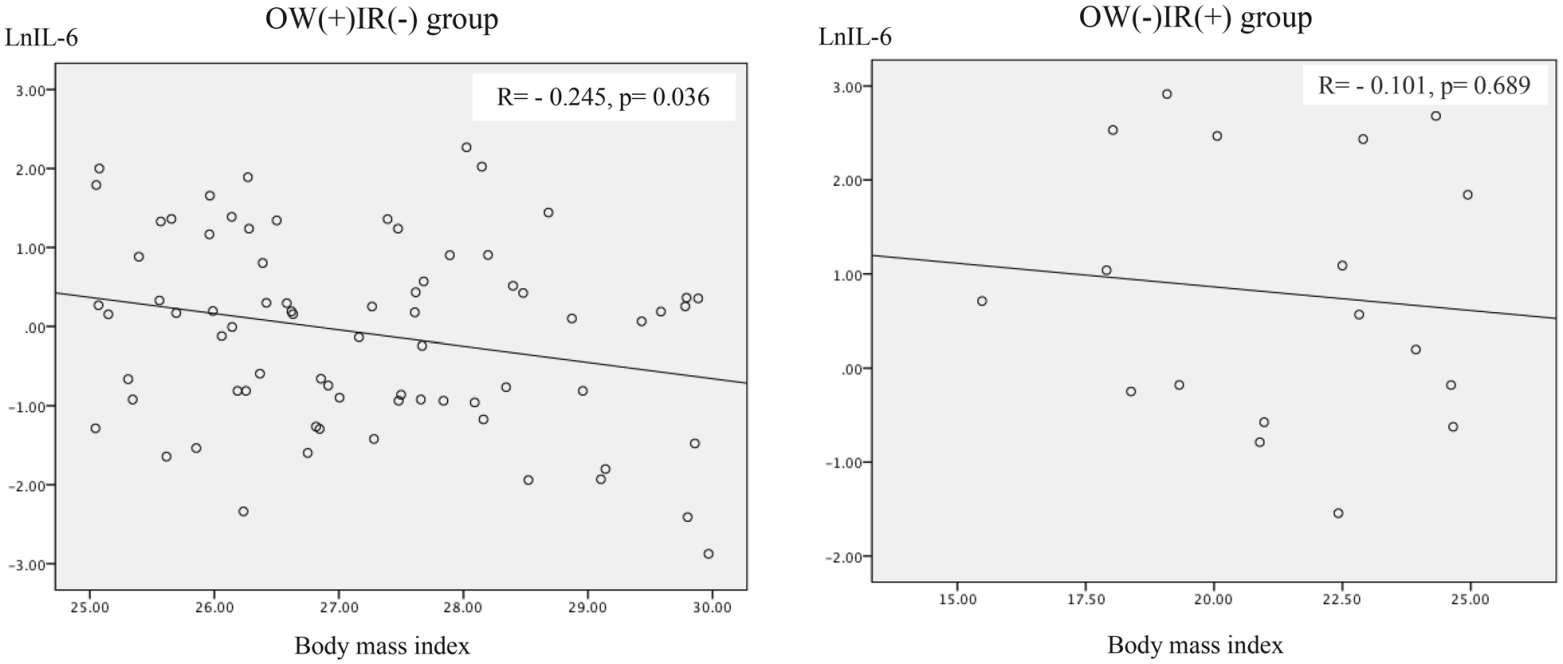
Correlation between interleukin-6 and body mass index, according to presence of overweight (n = 74) or insulin resistance (n = 18)

**Fig. 4:**
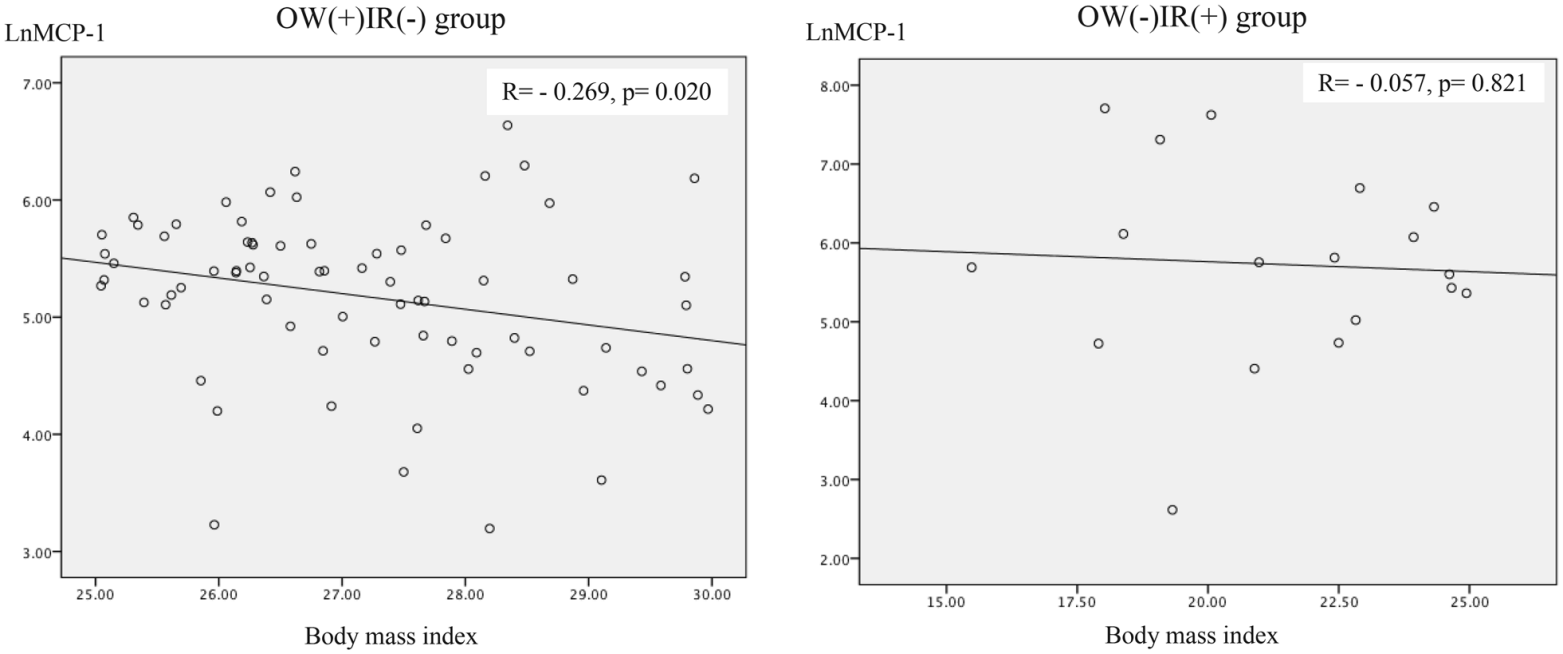
Correlation between MCP-1 and body mass index, according to presence of overweight (n = 74) or insulin resistance (n = 18)

In the OW(+)IR(−) group, the mean age was 39.2 ± 1.3, and 49 were female (66.2 %), and in the OW(−)IR(+) group, the mean age was 31.9 ± 3.6, and 12 were female (66.7 %). Demographic, anthropometric and biochemical variables are listed in Table 1. The variables that exhibited differences between the groups were age, skin color and high-risk abdominal circumference. The non-parametric analysis is shown in Table 2. Lean, insulin-resistant subjects [OW(−)IR(+) group] had significantly higher MCP-1 and IL-6 levels and a lower prevalence of high-risk waist circumference, compared with overweight non-insulin resistant group. Multivariate analysis by GEE showed that the presence of insulin resistance was associated with increased MCP-1 (OR = 1.005, p = 0.007) and IL-6 levels (OR = 1.263, p = 0.026) and with reduced adiponectin levels (OR = 0.893, p = 0.033) after adjustment for skin color, age, waist circumference and glycated hemoglobin (Table 3, model 1). In model 2 (Table 3), all of the studied cytokines were included, skin color and glycated hemoglobin were excluded. Model 2 (Table 3) shows that presence of insulin resistance was positively associated with MCP-1 (OR = 1.005, p = 0.024) and IL-6 levels; and, negatively associated with CRP levels (OR = 0.989, p = 0.049). In model 3 (Table 3), glycated hemoglobin was included, waist circumference was excluded. Model 3 (Table 3) shows that presence of insulin resistance was positively associated with resistin (OR = 1.017, p = 0.001), IL-6 (OR = 1.393, p = 0.002) and glycated hemoglobin (OR = 3.332, p = 0.036) and negatively associated with age (OR = 0.921, p = 0.024) and PAI-1 levels (OR = 0.981, p = 0.017). Waist circumference was included in model 4 (Table 3), in which it was observed that presence of insulin resistance was positively associated with MCP-1 (OR = 1.006, p = 0.024) and IL6 levels (OR = 1.361, p = 0.016), which were stronger than in the previous models 1 and 2. As shown in Figs. 1, 2, CRP levels presented a positive correlation with BMI (r = 0.695, p = 0.001) and waist circumference (r = 0.628, p = 0.005) in OW(−)IR(+) group. Conversely, in OW(+)IR(−) group, BMI presented negative correlation with IL-6 (r = −0.245, p = 0.036) and MCP-1 levels (r = −0.269, p = 0.020), as seen, respectively, in Figs. 3, 4.

**Table 1:** Demographic, anthropometric and laboratorial data, according to presence of overweight (n = 74) or insulin resistance (n = 18)

	OW(+)IR(−)	OW(−)IR(+)	p value
Gender			1,000
Female	49 (66.2)	12 (66.7)	
Male	25 (33.8)	6 (36.8)	
Age			<*0.001*
<20 years	1 (1.4)	7 (38.9)	
20–29	17 (23.0)	4 (22.2)	
30–39	19 (25.7)	1 (5.6)	
40–49	28 (37.8)	3 (16.7)	
50–59	7 (9.5)	3 (16.7)	
60 ou+	2 (2.7)	0 (5.3)	
Skin color			*0.016*
Black or mulatto	59 (78.7)	9 (50.0)	
White	15 (20.3)	9 (50.0)	
Total cholesterol			0.276
<200 mg/dL	46 (62.2)	14 (77.8)	
≥200 mg/dL	28 (37.8)	4 (22.2)	
HDL-C^a^			0.603
Normal	39 (52.7)	11 (61.1)	
Elevated	35 (47.3)	7 (38.9)	
Triglycerides			0.404
<150 mg/dL	67 (90.5)	15 (83.3)	
≥150 mg/dL	7 (9.5)	3 (16.7)	
Uric acid^b^			0.251
Normal	71 (95.9)	15 (88.9)	
Elevated	3 (4.1)	2 (11.1)	
Blood pressure			0. 529
Normal	59 (79.7)	13 (72.2)	
Hypertension	15 (20.3)	5 (27.8)	
Abdominal circumference			*0.027*
Normal	45 (60.8)	16 (88.9)	
High-risk	29 (39.2)	2 (11.1)	

^a^ Normal HDL-C: ≥40 mg/dL for men or ≥50 mg/dL for women

^b^ Elevated Uric Acid: >6.8 mg/dL for men and >6 mg/dL for women

Variables wtih *p* values <0.150 were selected for logistic binary modeling

Statistical significance was considered as *p* < 0.05 (in italics)

**Table 2:** Mann-Whitney Test for non-parametric variables, according to presence of overweight (n = 74) or insulin resistance (n = 18)

	OW(+)IR(−) (mean-rank)	OW(−)IR(+) (mean-rank)	P value
Age	49.88	32.61	*0.014*
*Circumference*			
Waist	53.05	19.58	*<0.001*
Abdominal	52.72	20.94	*<0.001*
Glycated hemoglobin	43.86	54.67	*0.120*
Cytokines			
C reactive protein	47.80	41.17	0.345
MCP-1^a^	43.34	59.50	*0.021*
IL-6^b^	43.42	59.17	*0.025*
Resistin	44.95	52.89	0.258
Adiponectin	43.95	56.97	*0.063*
PAI-1^c^	46.41	46.86	0.949

^a^Macrophage Chemoattractant Protein-1

^b^ Interleukin-6

^c^ Plasminogen activator inhibitor-1

Variables wtih *p* values <0.150 were selected for logistic binary modeling

Statistical significance was considered as *p* < 0.05 (in italics)

**Table 3:** Adjusted logistic regression models , according to presence of insulin resistance [OW(−)IR(+)] or absence [OW(+)IR(−)]

	Model 1	Model 2	Model 3	Model 4
	OR (95 %)	OR (95 %)	OR (95 %)	OR (95 %)
Age	1.083 (0.925–1.268)	1.064 (0.940–1.205)	0.921 (0.858–0.989)*	1.049 (0.898–1.225)
MCP-1^a^	1.005 (1.001–1.008)**	1.005 (1.001–1.010)*	1.003 (0.999–1.006)	1.006 (1.001–1.011)*
C reactive protein	*–*	0.989 (0.979–1.000)*	0.984 (0.966–1.003)	0.992 (0.976–1.008)
IL-6^b^	1.263 (1.029–1.551)*	1.361 (1.060–1.747)*	1.393 (1.133–1.713)**	1.390 (1.106–1.746)**
Resistin	–	1.016 (1.000–1.033)	1.017 (1.010–1.025)**	1.015 (0.997–1.034)
PAI-1^c^	–	0.993 (0.976–1.009)	0.981 (0.966–0.997)*	0.985 (0.961–1.011)
Adiponectin	0.893 (0.804–0.991)*	0.868 (0.727–1.037)	1.012 (0.978–1.048)	0.919 (0.741–1.139)
Waist circumf	0.653 (0.471–0.905)*	0.676 (0.514–0.888)**	–	0.693 (0.532–0.903)**
Glycated Hb	1.978 (0.813–4.814)	–	3.332 (1.079–10.293)*	2.575 (0.481–13.801)
White	0.457 (0.089–2.274)	–	–	–
Mulatto or black	1	–	–	–

Binary logistic model adjusted by GEE-In model 1, the variables included were that reached p value <0.150 on parametric and non-parametric tests (Tables 1 and 2). In model 2, all cytokines and a variable related to overweight were included. In model 3, all cytokines and a variable related to glucose metabolic imbalance were included, and waist circumference was excluded. In model 4, all cytokines, a variable related to glucose metabolic imbalance and a variable related to overweight were included

^a^ Macrophage Chemoattractant Protein-1

^b^ Interleukin-6

^c^ Plasminogen activator inhibitor-1

* p value <0.050

** p value <0.010

Statistical significance was considered as *p* < 0.05 (in italics)

## Discussion

In the present study, lean, insulin-resistant subjects presented a more inflammatory profile than overweight, insulin-sensitive subjects. Several studies have demonstrated that obesity promotes a chronic inflammatory state that is associated with inflammatory macrophage infiltration and accumulation in adipose tissue and that contributes to the development of insulin resistance and diabetes [12–15]. However, obesity *per se* is not a pre-condition for the development of insulin resistance but, rather, the degree of adipose tissue inflammation [4].

The local action of cytokines such as TNF-a on adipocytes was one of the first pieces of evidence suggesting a possible link between insulin resistance and pathogenic inflammatory processes [16]. Although adiposity is closely related to inflammation, our findings suggest that lean, insulin-resistant individuals could present higher levels of inflammatory biomarker even without excessive adipose tissue, compared with an overweight subject without insulin resistance. When endotoxemia was induced in healthy subjects, the acute inflammation was capable of modulating the signaling and inflammatory pathways in adipose tissue, thus causing insulin resistance, without an increase on adiposity [6]. Resistin is a pro-inflammatory cytokine that can promote systemic insulin resistance when injected in mice [1, 17]. Although this effect is supported by strong animal study evidences, the human-related data are less consistent [18, 19]. In our study, multivariate analysis indicated that lean, insulin-resistant subjects presented higher resistin levels (OR = 1.017, p = 0.001) compared with OW(+)IR(−) subjects after adjustment for age, IL-6, MCP-1, CRP, adiponectin and glycated hemoglobin levels (Table 3, model 3). The same association remained strong in models adjusted for waist circumference (Table 3, models 2, 4). Additionally, resistin-deficient ob/ob mice, even with an increased body adiposity, have normal glucose tolerance and preserved insulin sensitivity [20]. This suggests that resistin is a key player on the induction of insulin resistance and it is closely related to it.

MCP-1 is a cytokine that activates cells from the monocytic lineage, enhances CD11b/CD18 expression, and increases pro-inflammatory cytokines in human endotoxemia model [21, 22]. A recent study on transgenic mice demonstrated that MCP-1 overexpression in visceral adipose tissue resulted in elevated plasma MCP-1 levels and robust inflammatory macrophage recruitment that caused systemic insulin resistance [23]. Additionally, adipose tissue macrophages contribute significantly to the elevation of cytokine levels, such as TNF-a and IL-6 [13, 24]. IL-6 was one of the first pro-inflammatory cytokines to be implicated in insulin resistance pathogenesis and as a cardiovascular risk factor. Diabetic patients present high levels of serum IL-6 [25]. In our study, lean, insulin-resistant subjects had higher IL-6 (OR = 1.263, p = 0.026) and MCP-1 levels (OR = 1.005, p = 0.007) than OW(+)IR(−) patients (Table 3, model 1). After adjustment for age, waist circumference, resistin, CRP, PAI-1, adiponectin and glycated hemoglobin levels, the association of having insulin resistance and higher levels of IL-6 (OR = 1.390, p = 0.005) and MCP-1 (OR = 1.006, p = 0.026) became stronger (Table 3, model 4). Conversely, in an interesting Japanese study, increased visceral fat mass, as measured by tomography, was an independent predictor for the elevation of CRP levels in individuals with mild obesity or reduced glucose tolerance [26]. This is Japanese study also showed, with multiple linear regression, that visceral fat exhibited a higher correlation to CRP levels [26]. Similarly, in our present study, CRP levels had presented a strong correlation with BMI (r = 0.695, p = 0.001) and waist circumference (r = 0.628, p = 0.005) in the lean, insulin resistant group, as seen in Figs. 1, 2. However, these correlations were not found in the OW(+)IR(−) group. Additionally, the OW(+)IR(−) subjects had higher CRP levels according to logistic regression (OR = 0.989, p = 0.049) compared with the OW(−)IR(+) group, independent of age, waist circumference, IL-6, MCP-1, resistin, PAI-1 and adiponectin levels (Table 3, model 2).

The association between abdominal obesity and increased circulating PAI-1 levels was first described more than 20 years ago [27, 28]. More recently, it has been proposed that adipose tissue directly contributes to increased PAI-1 levels in obesity [29, 30]. Several interventional studies have demonstrated that a significant reduction in PAI-1 levels occurs after obese people lose weight through diet [31, 32] or jejunoileal bypass surgery [33]. Additionally, the reduced PAI-1 levels associated with losing weight is suggested to be related to the amount of weight lost and not to metabolic changes, such as variations on insulin or triglyceride levels [31, 32]. In our study, multivariate regression demonstrated that OW(+)IR(−) subjects had higher PAI-1 levels than the lean, insulin-resistant group (OR = 0.981, p = 0.017), independent of age, cytokine levels and glycated hemoglobin (Table 3, model 3). However, when the statistical models were adjusted for waist circumference, this association got weaker (Table 3, models 2, 4), which it is explained by the close relationship between PAI-1 and adiposity.

In contrast to other cytokines, adiponectin has anti-inflammatory, anti-apoptotic, and pro-angiogenic effects and is capable of enhancing insulin sensitivity on tissues [34, 35]. Reduced adiponectin levels are detected in diabetes, hypertension and coronary arterial disease, even when adjusted for BMI. Complementarily, insulin resistance diminished when hypoadiponectinemia is normalized by treatment with recombinant adiponectin in a type-2 diabetes animal model [36]. Interestingly, in a study of Pima Indians, who have high prevalence of type-2 diabetes, subjects with high adiponectin levels were found to be less likely to develop diabetes than those with low concentrations, which suggests a protective effect [37]. In our study, the multivariate regression revealed that the OW(+)IR(−) group had higher adiponectin levels compared with the OW(−)IR(+) group (OR = 0.893, p = 0.027), independent of age, waist circumference, skin color, IL-6, MCP-1, and glycated hemoglobin levels (Table 3, model 1). This finding is highly interesting because adiponectin levels are usually inversely related to BMI, waist circumference, body fat percentage and insulin resistance; however, in our study, adiponectin levels were higher in overweight subjects than in insulin-resistant subjects.

Obese or overweight but metabolically healthy individuals continue to represent a challenging phenotype. However, possible explanations reside in interpersonal differences in body fat distribution; life habits, such as physical activity; and metabolic dynamics of adipose tissue balance [38]. A recent study demonstrated that for the same degree of severe obesity, individuals with reduced adipose tissue inflammation exhibited an “intermediate” clinical phenotype with arterial function similar to that of normal weight subjects [39]. This evidence suggests that the exclusively overweight subjects (without insulin resistance) in our study represent a metabolically healthy phenotype with reduced inflammatory activity (lower cytokine and higher adiponectin levels) despite excess adipose tissue. Additionally, OW(+)IR(−) subjects had an inverse correlation of waist circumference with IL-6 levels (r = −0.245, p = 0.036). Cytokines that are closely related to adiposity (PAI-1 and CRP) were increased in the overweight group despite lower levels of the other cytokines (IL-6, MCP-1 and resistin) (Table 3).

The limitations of our study were its sample size and selection criteria; HOMA-IR was utilized to identify insulin resistance, although it is not the gold standard for diagnosis, and BMI, despite its prevalence, is not the most trusted index for estimating obesity. Although, BMI has a high and independent association with the risk of incidence of type II diabetes [40]. The impact of the latter is considered to be reduced by other adjusting factors, such as inflammatory markers, waist, abdominal and hip circumferences.

## Conclusion

Our findings suggest that an individual with normal BMI and a HOMA index greater than 2.6 has a significantly higher probability of presenting elevated levels of pro-inflammatory biomarkers (MCP-1, IL-6 and resistin) than an overweight subject with a HOMA index less than 2.6. More studies are necessary to clarify and extend the understanding of this complex syndrome.
